# Association of PCSK9 with inflammation and platelet activation markers and recurrent cardiovascular risks in STEMI patients undergoing primary PCI with or without diabetes

**DOI:** 10.1186/s12933-022-01519-3

**Published:** 2022-05-20

**Authors:** Li Song, Xiaoxiao Zhao, Runzhen Chen, Jiannan Li, Jinying Zhou, Chen Liu, Peng Zhou, Ying Wang, Yi Chen, Hanjun Zhao, Hongbing Yan

**Affiliations:** 1 Coronary Heart Disease Center, Fuwai Hospital, National Center for Cardiovascular Diseases, Chinese Academy of Medical Science and Peking Union Medical College, No. 167, Beilishi Road, Xicheng District, 100037 Beijing, China; 2 Fuwai Hospital, Chinese Academy of Medical Sciences, 12 Langshan Rd, Shenzhen, 518000 China

**Keywords:** PCSK9, Metabolism, Inflammation, Platelet activation, Diabetes mellitus, STEMI, Percutaneous coronary intervention, MACEs

## Abstract

**Background:**

Proprotein convertase subtilisin/kexin type 9 (PCSK9) has been shown to be predictive of cardiovascular outcomes in stable coronary artery disease with diabetes. We aimed to assess the relationship between PCSK9 and major adverse cardiovascular events (MACEs) in ST-segment elevation myocardial infarction (STEMI) patients with or without diabetes, as well as the relationships between PCSK9 and metabolism, inflammation and platelet activation markers.

**Methods:**

A total of 1027 patients with STEMI undergoing primary percutaneous coronary intervention (PCI) and without prior lipid-lowering therapy were consecutively enrolled and the baseline plasma PCSK9 levels were determined by ELISA. Patients were divided into high and low PCSK9 groups according to PCSK9 median. All patients were followed up for the occurrence of MACEs. The associations of PCSK9 with metabolism, inflammation and platelet activation markers and MACEs were evaluated.

**Results:**

PCSK9 levels were positively correlated with triglycerides, high-sensitivity C reactive protein, soluble CD40 ligand and soluble P-selectin levels, and the correlations were stronger in diabetic patients than in non-diabetic patients. In diabetic patients receiving ticagrelor, PCSK9 levels were positively correlated with maximal platelet aggregation measured by light transmittance aggregometry and maximum amplitude of adenosine diphosphate-induced platelet-fibrin clots measured by thrombelastography in the maintenance phase of treatment, whereas no correlations were found in non-diabetic patients. During a median follow-up of 2.0 years, 155 (15.1%) MACEs occurred. The Kaplan–Meier analysis displayed that the patients with high PCSK9 levels had lower event-free survival rate than those with low PCSK9 levels (P = 0.030). When participants were categorized into 4 subgroups according to PCSK9 levels and diabetes status, high PCSK9 levels plus diabetes subgroup had the lowest cumulative event-free survival rate (P = 0.043). Multivariable Cox regression analysis revealed that high PCSK9 levels were independently associated with MACEs in diabetic patients (hazard ratio 2.283, 95% confidence interval: 1.094–4.764, P = 0.028), but not in the whole cohort or non-diabetic patients.

**Conclusions:**

The study showed that high PCSK9 levels were independently associated with MACEs in STEMI patients with diabetes undergoing primary PCI, and the association may be due to stronger correlations of PCSK9 with inflammation and platelet activation markers in diabetic patients.

**Supplementary Information:**

The online version contains supplementary material available at 10.1186/s12933-022-01519-3.

## Background

The risk of recurrent cardiovascular events is still high in patients with acute coronary syndrome (ACS) despite the advancements of interventional and pharmacologic strategies. Several large-scale trials have demonstrated that human proprotein convertase subtilisin/kexin type 9 (PCSK9) inhibitors markedly reduce low-density lipoprotein cholesterol (LDL-C) levels and future cardiovascular events [1, 2]. Furthermore, a growing number of studies suggest that circulating PCSK9 might exert adverse effects on cardiovascular system through several pathways beyond LDL-C regulation, such as promoting inflammatory response and increasing platelet activation [3–5]. Consequently, circulating PCSK9 concentration has been proposed to be a novel biomarker for predicting major adverse cardiovascular events (MACEs) in coronary artery disease (CAD) [6]. However, the association between PCSK9 and MACEs in ACS remains unclear, given inconsistent results [7–10]. A recent study showed that the baseline PCSK9 levels were independently associated with the risk of MACEs in stable CAD patients with diabetes mellitus (DM), and the patients with high PCSK9 levels plus DM had an extremely high risk of MACEs compared with those with low PCSK9 levels and non-DM [11]. However, no study has yet determined the association between PCSK9 and MACEs in ACS patients with DM.

Therefore, the aims of this study were to assess: (1) the relationship between baseline PCSK9 levels and incidence of MACEs in patients with ST-segment elevation myocardial infarction (STEMI) undergoing primary percutaneous coronary intervention (PCI) with or without DM; and (2) the relationships between PCSK9 levels and metabolism, inflammation and platelet activation markers.

## Methods

### Study population

**Fig. 1:**
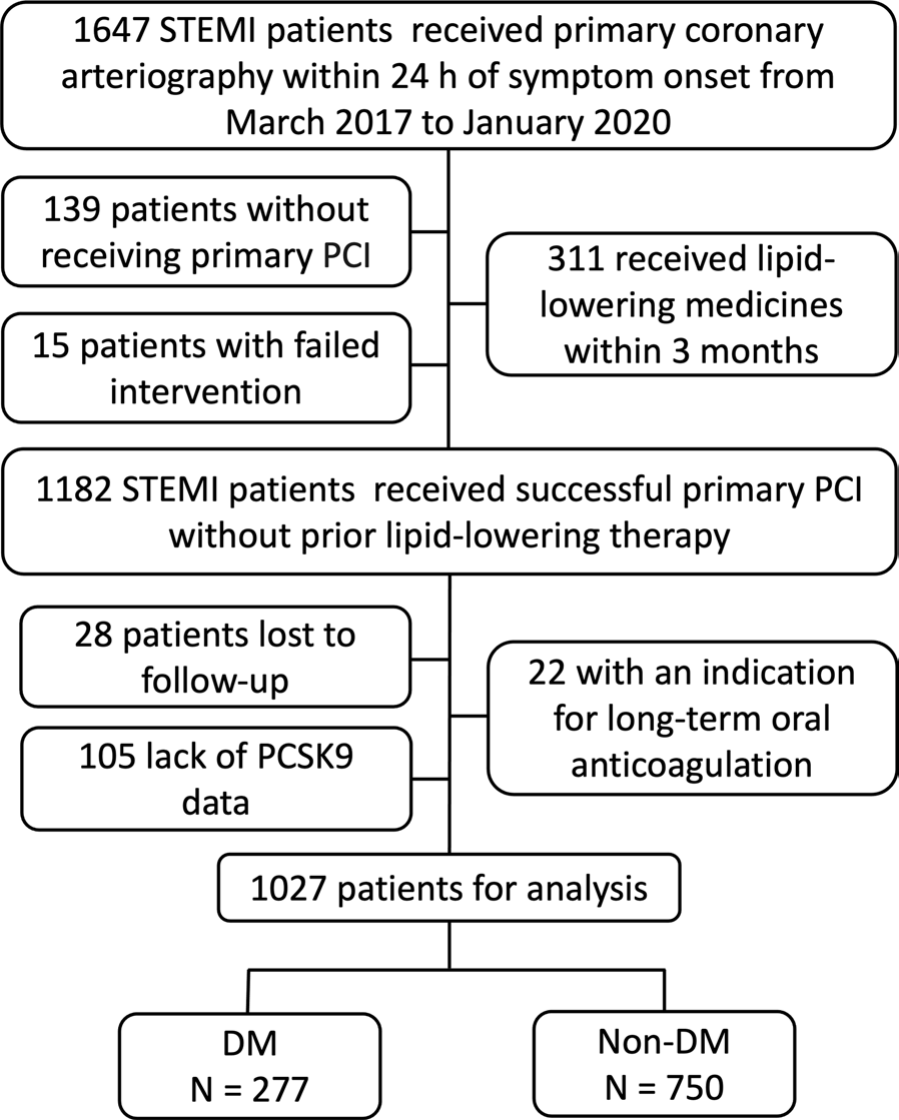
Study flow chart. *STEMI* ST-segment elevation myocardial infarction, *PCI* percutaneous coronary intervention, *PCSK9* proprotein convertase subtilisin/kexin type 9, *DM* diabetes mellitus, *Non-DM* non-diabetes mellitus

This was a single-center observational study. As shown in Fig. 1, from March 2017 to January 2020, a total of 1647 adult patients (18 years or older) presenting with STEMI who underwent primary coronary arteriography within 24 h of symptom onset were consecutively screened for enrolment. The definition of STEMI followed the established criteria [12]. For this analysis, we excluded 139 patients without receiving PCI, 15 patients with failed intervention, 311 patients receiving lipid-lowering medicines within 3 months before admission, 105 patients lack of PCSK9 data, 22 patients with an indication for long-term oral anticoagulation as well as 28 patients who were lost to follow-up. Finally, 1027 cases undergoing successful primary PCI and without prior lipid-lowering therapy were included. Diagnosis of DM was based on one of the following criteria: self-reported DM, fasting plasma glucose level ≥ 7.0 mmol/L (126 mg/dL), fasting plasma HbA1C level ≥ 6.5%, or treated with oral hypoglycemic agents or insulin.

All participants were treated with aspirin, a P2Y_12_ inhibitor (ticagrelor or clopidogrel) and other standard therapies including statin according to guidelines and at the discretion of the treating physicians. Ticagrelor or clopidogrel was provided with a loading dose of 180 mg or 300 to 600 mg and a maintenance dose of 90 mg twice daily or 75 mg daily, respectively. None of the patients received PCSK9 inhibitors.

The study protocol was carried out in accordance with the Declaration of Helsinki and approved by the Ethics Review Board of Fuwai Hospital & National Center for Cardiovascular Diseases (approval number 2017-866). All patients provided written informed consent.

### Blood sample measurement

Baseline blood samples for measurement of PCSK9, soluble CD40 ligand (sCD40L) and soluble P-selectin (sP-selectin) were immediately processed at admission by centrifugation at 2000×*g* for 15 min at room temperature and the isolated plasma was frozen at − 80 ℃ until use. Plasma concentrations of PCSK9, sCD40L and sP-selectin were measured using enzyme-linked immunosorbent assay (ELISA) (DY3888, DY617 and DY137, respectively; R&D Systems; Catalog) according to the manufacturer’s instructions. We have conducted a more detailed protocol and deposited it as Additional file 1.

Additional venous blood samples were collected at admission immediately for measurements of other laboratory parameters (including complete blood cell count, high-sensitivity C reactive protein [hs-CRP], creatinine, and fibrinogen), or after a 10–12 h overnight fasting for metabolic parameters (including lipids profiles, glucose and HbA1C). All samples were tested by standard laboratory techniques at the Center of Laboratory Medicine of the Fuwai hospital.

Platelet reactivity was assessed by light transmittance aggregometry (LTA) and thrombelastography (TEG) in the morning before a maintenance dose of ticagrelor or clopidogrel (i.e., trough level) at 7 days post-PCI. Blood samples for LTA measurement were drawn into vacutainer tubes containing 0.5 mL of sodium citrate 3.2% (Becton-Dickinson, San Jose, CA, USA) and processed within 2 h according to standard operating procedures. Platelet-rich plasma was obtained as a supernatant after centrifuging the blood at 120×*g* for 5 min. The remaining blood was further centrifuged at 1200 × g for 10 min to obtain platelet‑poor plasma. Platelet aggregation was assessed at 37 °C with an AggRam aggregometer (Helena Laboratories, Corp., Beaumont, TX, USA). Platelets were stimulated with 5 µmol/L adenosine diphosphate (ADP), and platelet reactivity values are reported as percentages of maximal platelet aggregation (MPA). Blood samples for TEG measurement were processed within 2 h after blood draw according to standard procedures. The maximum amplitude of ADP-induced platelet-fibrin clots (MA_ADP_) was measured with a TEG hemostasis system (Haemoscope Corp., Massachusetts, USA).

### Clinical outcomes and follow-up

The primary outcome was a combined endpoint of MACEs, including all-cause death, recurrent myocardial infarction (MI), ischemic stroke and rehospitalization for heart failure. MI was defined in accordance with the universal definition [13]. Ischemic stroke was confirmed by a neurologist on the basis of imaging studies and was defined as a new neurologic deficit lasting > 24 h, and computed tomography or magnetic resonance imaging was performed to verify acute cerebral infarction. Follow-up was performed by well-trained physicians routinely at 1, 6, and 12 months after discharge and every 6 months thereafter via direct interviews, telephone calls and discharge records or clinical notes. There are two professional physicians blinded to the clinical and laboratory data confirmed the follow-up endpoints.

### Statistical analysis

The statistical analyses were performed using SPSS software, version 25 (IBM, Armonk, NY). Distribution of continuous variables was assessed according to the Kolmogorov-Smirnov test. Continuous data are presented as mean ± standard deviation (SD) for normally distributed data or median (interquartile range) for non-normally distributed data. Between group differences were tested using the independent sample t-test or the Mann–Whitney U test. Categorical data are presented as counts (percentage), and were compared using Pearson’s χ2 test. Spearman correlation analyses were used to evaluate the correlations of PCSK9 levels with metabolism, inflammation and platelet activation and reactivity parameters. The cumulative event-free survival rates among subgroups according to PCSK9 levels or/and DM status were examined by the Kaplan–Meier curves with the log-rank test. Univariate and multivariate Cox regression analyses were used to calculate the hazard ratios (HR) of adverse cardiovascular events with 95% confidence intervals (CI). Receiver operating characteristic (ROC) curves were plotted to examine the sensitivity, specificity and area under the curves (AUC) of PCSK9 for predicting MACEs among patients in the whole cohort, DM patients and non-DM patients. The cut-off values were determined using Youden index. The accuracy of risk models of established risk factors with or without PCSK9 for predicting MACEs among patients in the whole cohort, DM patients and non-DM patients was also assessed by area under the ROC curves, and compared using a nonparametric test developed by DeLong et al. with the use of MedCalc software for Windows, version18.2.1 (MedCalc Software, Ostend, Belgium). A two-tailed P value of < 0.05 was considered statistically significant.

## Results

### Baseline characteristics

The mean age of this population was 59.6 ± 12.7 years, 81.2% were male, and 277 (27.0%) suffered from DM. Patient characteristics according to the occurrence of MACEs and the median of PCSK9 concentration are detailed in Table 1. The interquartile PCSK9 ranged from 24.1 to 83.8 ng/mL, with a median of 43.5 ng/mL.

**Table 1:** Baseline characteristics of patients according to MACEs and PCSK9 stratification

Variables	Whole cohort (n = 1027)	MACEs		P value	PCSK9 (ng/mL)		P value
		No (n = 872)	Yes (n = 155)		≤ 43.5 (n = 514)	> 43.5 (n = 513)	
*Demographics*							
Age, years	59.6 ± 12.7	58.9 ± 12.5	63.8 ± 13.1	< 0.001	59.8 ± 12.2	59.5 ± 13.2	0.685
Male, %	834 (81.2)	718 (82.3)	116 (74.8)	0.028	431 (83.9)	403 (78.6)	0.030
Body mass index, kg/m^2^	26.1 ± 3.8	26.2 ± 3.7	25.6 ± 4.0	0.067	25.9 ± 3.5	26.2 ± 4.0	0.290
*Risk factors*							
Hypertension	602 (58.6)	495 (56.8)	107 (69.0)	0.004	302 (58.8)	300 (58.5)	0.929
Hyperlipidemia	754 (73.4)	643 (73.7)	111 (71.6)	0.581	380 (73.9)	374 (72.9)	0.710
Diabetes mellitus	277 (27.0)	231 (26.5)	46 (29.7)	0.410	136 (26.5)	141 (27.5)	0.711
Current smoking	529 (51.5)	463 (53.1)	66 (42.6)	0.016	267 (51.9)	262 (51.1)	0.779
Familial history of CAD	210 (20.4)	179 (20.5)	31 (20.0)	0.881	103 (20.0)	107 (20.9)	0.745
*Clinical and angiographic characteristics*							
Anterior infarction	487 (47.4)	406 (46.6)	81 (52.3)	0.190	226 (44.0)	261 (50.9)	0.027
Systolic blood pressure, mmHg	130.6 ± 22.6	131.0 ± 22.2	128.7 ± 24.5	0.251	130.1 ± 21.8	131.2 ± 23.4	0.397
LVEF at admission	53.5 ± 7.5	54.2 ± 7.0	49.7 ± 8.7	< 0.001	54.1 ± 7.0	53.0 ± 7.9	0.028
TIMI score	3.6 ± 2.1	3.4 ± 2.0	4.6 ± 2.8	< 0.001	3.6 ± 2.1	3.6 ± 2.1	0.694
Symptom-to-door, mins	300.0 (150.0, 628.5)	300.0 (150.0, 624.0)	285.0 (165.0, 660.0)	0.944	300.0 (178.5, 606.0)	279.0 (123.3, 658.8)	0.453
Door-to-procedure, mins	111.0 (85.0, 152.0)	111.0 (84.0, 153.0)	114.0 (89.0, 148.5)	0.598	114.0 (86.0, 156.5)	111.0 (84.0, 148.5)	0.521
Symptom-to-procedure, mins	431.0 (260.3, 791.8)	434.0 (260.0, 784.3)	407.5 (263.3, 796.3)	0.816	443.5 (279.8, 798.8)	406.5 (243.8, 784.8)	0.375
Coronary artery lesions							
Single vessel disease	300 (29.2)	269 (30.8)	31 (20.0)	0.018	152 (29.6)	148 (28.8)	0.545
Double vessel disease	303 (29.5)	255 (29.2)	48 (31.0)		158 (30.7)	145 (28.3)	
Triple vessel disease	424 (41.3)	348 (39.9)	76 (49.0)		204 (39.7)	220 (42.9)	
Pre-TIMI flow							
0	658 (64.1)	554 (63.5)	104 (67.1)	0.117	331 (64.4)	327 (63.7)	0.581
1	38 (3.7)	28 (3.2)	10 (6.5)		15 (2.9)	23 (4.5)	
2	96 (9.3)	84 (9.6)	12 (7.7)		47 (9.1)	49 (9.6)	
3	235 (22.9)	206 (23.6)	29 (18.7)		121 (23.5)	114 (22.2)	
AHA classification							
A	19 (1.9)	16 (1.8)	3 (1.9)	0.314	8 (1.6)	11 (2.1)	0.240
B1	102 (9.9)	86 (9.9)	16 (10.3)		42 (8.3)	60 (11.6)	
B2	210 (20.4)	187 (21.4)	23 (14.8)		102 (20.0)	108 (20.8)	
C	696 (67.8)	583 (66.9)	113 (72.9)		357 (70.1)	339 (65.4)	
*Laboratory examinations*							
Total cholesterol, mg/dL	175.8 ± 38.6	176.8 ± 37.9	170.2 ± 41.9	0.056	175.0 ± 36.3	176.6 ± 40.7	0.507
LDL-C, mg/dL	112.2 ± 32.5	112.8 ± 31.9	108.6 ± 35.2	0.144	111.9 ± 31.2	112.5 ± 33.7	0.761
HDL-C, mg/dL	42.0 ± 11.4	42.0 ± 11.0	42.2 ± 13.4	0.685	43.1 ± 12.3	40.9 ± 10.4	0.002
Triglycerides, mg/dL	154.4 ± 107.5	155.4 ± 107.2	148.6 ± 109.1	0.474	144.7 ± 97.6	164.1 ± 115.7	0.004
Lipoprotein(a), g/L	161.0 (74.0, 338.2)	161.0 (75.5, 338.0)	158.1 (63.8, 341.0)	0.299	148.5 (68.0, 338.8)	177.0 (80.2, 338.6)	0.061
HbA1C, %	6.7 ± 1.6	6.6 ± 1.6	6.9 ± 1.8	0.073	6.6 ± 1.6	6.7 ± 1.6	0.164
Fasting plasma glucose	8.5 ± 3.9	8.4 ± 3.7	9.0 ± 4.5	0.103	8.5 ± 3.8	8.6 ± 3.9	0.705
Leukocytes, ×1000/µL	10.6 ± 3.2	10.5 ± 3.1	11.1 ± 3.7	0.026	10.5 ± 3.2	10.7 ± 3.2	0.221
Hemoglobin, g/L	147.3 ± 17.6	147.9 ± 17.6	144.1 ± 17.5	0.012	147.6 ± 16.5	147.1 ± 18.6	0.670
Platelet, ×1000/µL	234.8 ± 72.7	233.1 ± 74.2	244.0 ± 62.5	0.087	233.0 ± 79.4	236.6 ± 65.3	0.437
eGFR, mL/min/1.73 m^2^	92.2 ± 31.7	94.0 ± 31.0	81.9 ± 33.8	< 0.001	93.6 ± 30.9	90.8 ± 32.5	0.156
Fibrinogen, µmol/L	3.8 ± 2.2	3.8 ± 2.2	4.2 ± 2.0	0.021	3.8 ± 2.8	3.9 ± 1.3	0.231
hs-CRP, mg/L	6.5 (2.5, 11.0)	6.2 (2.3, 10.9)	8.8 (3.3, 11.5)	0.007	5.6 (2.2, 10.6)	7.5 (2.7, 11.2)	0.003
PCSK9, ng/mL	43.5 (24.1, 83.8)	41.8 (24.1, 80.6)	51.8 (24.7, 93.5)	0.018	24.2 (13.5, 32.7)	83.8 (59.3, 126.1)	< 0.001
*Medication at discharge*							
Aspirin	1019 (99.2)	866 (99.3)	153 (98.7)	0.432	510 (99.2)	509 (99.2)	1.000
Ticagrelor	563 (54.8)	488 (56.0)	75 (48.4)	0.081	292 (56.8)	271 (52.8)	0.200
Clopidogrel	464 (45.2)	384 (44.0)	80 (51.6)	0.081	222 (43.2)	242 (47.2)	0.200
Statin	988 (96.2)	841 (96.4)	147 (94.8)	0.335	493 (95.9)	495 (96.5)	0.629
Beta-Blockers	885 (86.2)	755 (86.6)	130 (83.9)	0.367	445 (86.6)	440 (85.8)	0.708
Renin angiotensin system inhibitor	736 (71.7)	637 (73.1)	99 (63.9)	0.019	377 (73.3)	359 (70.0)	0.231

Data are presented as mean ± SD or number (%) or median (interquartile range). *MACEs* major adverse cardiac events (including all-cause death, recurrent myocardial infarction, ischemic stroke and rehospitalization for heart failure), *PCSK9* proprotein convertase subtilisin/kexin type 9, *CAD* coronary artery disease, *LVEF* left ventricular ejection fraction, *TIMI* thrombolysis in myocardial infarction, *AHA* American Heart Association, *LDL-C* low-density lipoprotein cholesterol, *HDL-C* high-density lipoprotein cholesterol, *HbA1C* hemoglobin A1c, *eGFR* estimated glomerular filtration rate, *hs-CRP* high-sensitivity C reactive protein

As shown in Table 1, patients with MACEs were older and less likely to be male or current smoker, had a higher prevalence of hypertension, and presented with more multi-vessel or left main disease (all P < 0.05). In addition, patients with MACEs tend to have higher TIMI score, leukocyte counts, fibrinogen and hs-CRP, but lower estimated glomerular filtration rate, hemoglobin and left ventricular ejection fraction (LVEF) (all P < 0.05). As expected, patients with MACEs had higher PCSK9 levels compared to those without [51.8 (24.7, 93.5) vs. 41.8 (24.1, 80.6) ng/mL, P = 0.018]. However, the prevalence of DM was not different between patients with and without events.

Patients with higher PCSK9 levels were more likely to be female, presented with more frequent anterior infarction, and had higher triglycerides and hs-CRP and lower LVEF and high-density lipoprotein cholesterol (HDL-C) (all P < 0.05). The prevalence of DM was not different between patients with higher and lower PCSK9 levels.

Among discharge medications, angiotensin-converting enzyme inhibitors or angiotensin receptor blockers were more frequently used in patients with MACEs compared to those without these events (73.1% vs. 63.9%, P = 0.019). However, no differences were detected regarding the medications between patients with higher and lower PCSK9 levels.

### Associations between PCSK9 levels and metabolism, inflammation and platelet activation and reactivity markers

PCSK9 levels were negatively associated with fasting glucose in DM patients (r = − 0.146, P = 0.031), whereas no correlations were found in the whole cohort and non-DM patients. Regarding lipid metabolic parameters, PCSK9 levels were positively associated with triglycerides in the whole cohort (r = 0.146, P < 0.001), and the correlation was stronger in DM patients than in non-DM patients (r = 0.214, P < 0.001; r = 0.111, P = 0.003; respectively). Moreover, PCSK9 levels were negatively associated with HDL-C and positively associated with lipoprotein(a) in the whole cohort and non-DM patients, while the strength of the correlations was weak. However, no significant correlations were found between PCSK9 levels and other metabolic parameters including body mass index, HbA1C, total cholesterol and LDL-C (Table 2).

**Table 2:** Spearman correlation analyses between PCSK9 and metabolism and inflammation makers according to diabetes status

	Whole cohort (n = 1027)		DM (n = 277)		Non-DM (n = 750)	
	r	P	r	P	r	P
Metabolism related markers						
Body mass index	0.021	0.507	− 0.020	0.747	0.034	0.355
Fasting glucose	− 0.038	0.229	− 0.133	0.031	− 0.018	0.622
HbA1C	0.040	0.207	− 0.048	0.438	0.062	0.096
Total cholesterol	0.044	0.164	0.038	0.536	0.047	0.210
LDL-C	0.005	0.872	− 0.039	0.525	0.022	0.549
HDL-C	− 0.081	0.011	0.023	0.706	− 0.120	0.001
Triglycerides	0.146	< 0.001	0.214	< 0.001	0.111	0.003
Lipoprotein(a)	0.075	0.019	0.048	0.438	0.089	0.017
Inflammatory marker						
hs-CRP	0.112	< 0.001	0.205	0.001	0.076	0.041

*PCSK9* proprotein convertase subtilisin/kexin type 9, *DM* diabetes mellitus, *non-DM* non-diabetes mellitus, *HbA1C* hemoglobin A1c, *LDL-C* low-density lipoprotein cholesterol, *HDL-C* high-density lipoprotein cholesterol, *hs-CRP* high-sensitivity C reactive protein, *sCD40L* soluble CD40 ligand

Regarding inflammatory maker, PCSK9 levels were significantly and positively associated with hs-CRP in the whole cohort (r = 0.112, P < 0.001), and the correlation was stronger in DM patients (r = 0.205, P = 0.001) than in non-DM patients (r = 0.076, P = 0.041) (Table 2).

Regarding platelet activation makers, PCSK9 levels were significantly and positively associated with sP-selectin and sCD40L in the whole cohort (r = 0.158, P = 0.015; r = 0.176, P < 0.001; respectively), and the correlations were stronger in DM patients (r = 0.181, P = 0.001; r = 0.279, P < 0.001; respectively) than in non-DM patients (r = 0.141, P = 0.065; r = 0.086, P = 0.001; respectively). The results were consistent after excluding 130 patients who had taken antiplatelet drugs for more than 2 weeks before admission (Table 3).

**Table 3:** Spearman correlation analyses between PCSK9 and platelet activation makers according to diabetes status

	Whole cohort (n = 1027)		DM (n = 277)		Non-DM (n = 750)	
	r	P	r	P	r	P
sP-selectin	0.158	0.015	0.181	0.001	0.141	0.065
sCD40L	0.176	< 0.001	0.279	< 0.001	0.086	0.001
Without prior antiplatelet therapy (n = 897)			n = 225		n = 672	
sP-selectin	0.133	0.012	0.216	< 0.001	0.105	0.057
sCD40L	0.219	< 0.001	0.317	< 0.001	0.187	< 0.001

*PCSK9* proprotein convertase subtilisin/kexin type 9, *DM* diabetes mellitus, *non-DM* non-diabetes mellitus, *sP-selectin* soluble P-selectin, *sCD40L* soluble CD40 ligand

In DM patients receiving ticagrelor, PCSK9 levels were significantly and positively associated with MPA and MA_ADP_ at 7 days post-PCI (r = 0.236, P = 0.041; r = 0.336, P = 0.013; respectively), whereas no correlations were found in non-DM patients receiving ticagrelor. Additionally, there were no correlations of PCSK9 levels with platelet reactivity parameters in those treated with clopidogrel regardless of DM status (Table 4).

**Table 4:** Spearman correlation analyses between PCSK9 and platelet reactivity parameters according to diabetes status and P2Y_12_ inhibitor stratification

	Whole cohort (n = 1027)		DM (n = 277)		Non-DM (n = 750)	
	r	P	r	P	r	P
Ticagrelor	n = 563		n = 143		n = 420	
MPA at 7 days	0.144	0.012	0.236	0.041	0.111	0.094
MA_ADP_ at 7 days	0.080	0.228	0.336	0.013	0.033	0.664
Clopidogrel	n = 464		n = 134		n = 330	
MPA at 7 days	0.100	0.113	− 0.078	0.586	0.190	0.110
MA_ADP_ at 7 days	− 0.048	0.546	− 0.172	0.160	− 0.041	0.667

*PCSK9* proprotein convertase subtilisin/kexin type 9, *DM* diabetes mellitus, *Non-DM* non-diabetes mellitus, *MPA* maximal platelet aggregation, *MA*_*ADP*_ maximum amplitude of adenosine diphosphate-induced platelet-fibrin clots

### Associations between PCSK9 levels and clinical outcomes

**Fig. 2:**
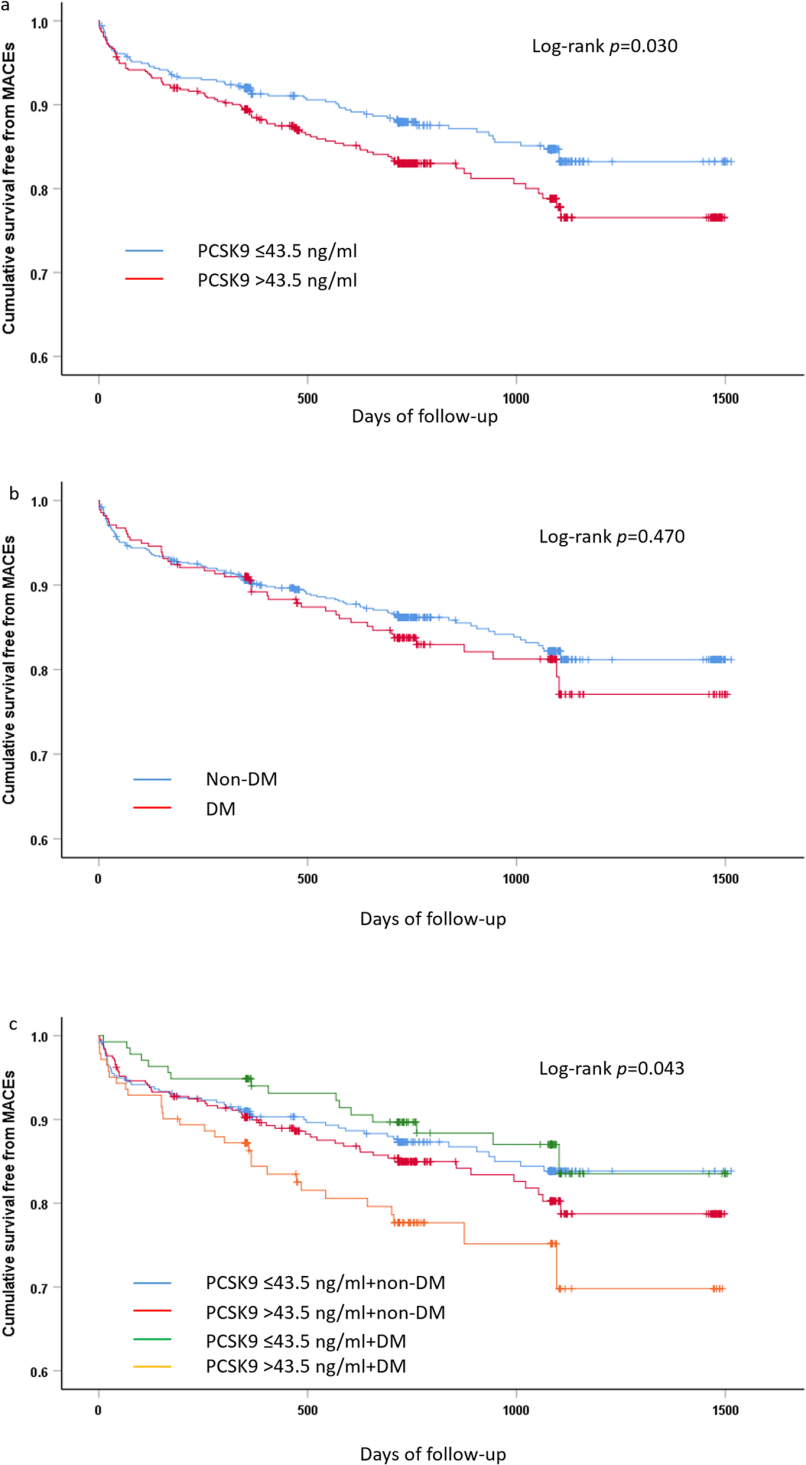
Kaplan–Meier analysis according to different PCSK9 levels (**a**), diabetic status (**b**) and status of both PCSK9 levels and diabetic status (**c**). *PCSK9* proprotein convertase subtilisin/kexin type 9, *Non-DM* non-diabetes mellitus, *DM* diabetes mellitus

During a median follow-up of 2.0 years (interquartile range, 1.3 to 3.0 years), 155 (15.1%) MACEs occurred. Of these, there were 62 all-cause death, 41 recurrent MI, 37 ischemic stroke, and 23 rehospitalization for heart failure. Kaplan–Meier curve analysis displayed that the patients with high PCSK9 levels had lower event-free survival rate compared to those with low PCSK9 levels in the whole cohort (P = 0.030, Fig. 2a). Although DM patients were more likely to suffer from MACEs compared with those without DM, the difference was not significant (P = 0.470, Fig. 2b). When participants were further categorized into 4 subgroups according to PCSK9 levels and DM status, the high PCSK9 levels plus DM subgroup had the lowest cumulative event-free survival rate among the four subgroups (P = 0.043, Fig. 2c).

As presented in Table 5, univariate Cox regression analysis revealed a significant association between high PCSK9 levels and increased risk of MACEs or rehospitalization for heart failure in the whole cohort (HR: 1.420, 95% CI 1.033–1.953, P = 0.031; HR: 2.463, 95% CI 1.011–5.988, P = 0.047; respectively). In addition, univariate analysis showed that high PCSK9 levels were associated with an increased risk of MACEs, all-cause death or recurrent MI in DM subjects. However, after adjusting for confounding factors, only a significant association between high PCSK9 levels and increased risk of MACEs in DM subjects was observed (HR: 2.283, 95% CI 1.094–4.764, P = 0.028) (Table 5 and Additional file 2: Table S1). Further, multivariate Cox regression analysis based on the combination of PCSK9 levels and DM status revealed that only the subgroup with DM plus high PCSK9 levels had a significantly higher risk of MACEs compared with the reference subgroup (non-DM plus low PCSK9 levels) (HR: 1.996, 95% CI 1.047–3.817, P = 0.036) (Additional file 2: Table S2).

**Table 5:** Multivariable Cox regression analyses of PCSK9 levels for clinical outcomes

Events	PCSK9 (ng/mL)		Unadjusted		Multivariate adjusted*	
	≤ 43.5	> 43.5	HR (95% CI)	P	HR (95% CI)	P
Whole cohort	n = 514	n = 513				
MACEs	67 (13.0)	88 (17.2)	1.420 (1.033–1.953)	0.031	1.361 (0.964–1.923)	0.080
All-cause death	26 (5.1)	36 (7.0)	1.443 (0.870–2.392)	0.155	1.312 (0.730–2.358)	0.364
Recurrent myocardial infarction	18 (3.5)	23 (4.5)	1.364 (0.736–2.532)	0.324	1.357 (0.720–2.558)	0.345
Ischemic stroke	17 (3.3)	20 (3.9)	1.305 (0.683-2.500)	0.419	1.302 (0.668–2.538)	0.437
Rehospitalization for heart failure	7 (1.4)	16 (3.1)	2.463 (1.011–5.988)	0.047	1.550 (0.597–4.032)	0.368
DM	n = 136	n = 141				
MACEs	16 (11.8)	30 (21.3)	2.200 (1.195–4.048)	0.011	2.283 (1.094–4.764)	0.028
All-cause death	5 (3.7)	14 (9.9)	3.021 (1.086–8.403)	0.034	2.646 (0.774–9.091)	0.121
Recurrent myocardial infarction	1 (0.7)	8 (5.7)	9.174 (1.134–71.429)	0.038	-	–
Ischemic stroke	9 (6.6)	8 (5.7)	1.017 (0.391–2.646)	0.972	1.004 (0.351–2.873)	0.993
Rehospitalization for heart failure	1 (0.7)	3 (2.1)	3.802 (0.387–37.037)	0.252	–	–
Non-DM	n = 378	n = 372				
MACEs	51 (13.5)	58 (15.6)	1.207 (0.828–1.759)	0.329	1.145 (0.764–1.718)	0.512
All-cause death	21 (5.6)	22 (5.9)	1.091 (0.560–1.984)	0.776	1.125 (0.548–2.257)	0.769
Recurrent myocardial infarction	17 (4.5)	15 (4.0)	0.926 (0.462–1.855)	0.828	0.926 (0.454–1.883)	0.829
Ischemic stroke	8 (2.1)	12 (3.2)	1.650 (0.674–4.049)	0.273	1.742 (0.694–4.367)	0.237
Rehospitalization for heart failure	6 (1.6)	13 (3.5)	2.273 (0.863–5.988)	0.097	1.748 (0.617–4.950)	0.293

*PCSK9* proprotein convertase subtilisin/kexin type 9, *HR* hazard ratio, *CI* confidence interval, *MACEs* major adverse cardiac events (including all-cause death, recurrent myocardial infarction, ischemic stroke and rehospitalization for heart failure), *DM* diabetes mellitus, *Non-DM* non-diabetes mellitus. *Adjusted for age, sex, body mass index, hypertension, smoking status, left ventricular ejection fraction, thrombolysis in myocardial infarction score, coronary artery lesions, total cholesterol, HbA1c, leukocyte count, hemoglobin, estimated glomerular filtration rate, high-sensitivity C reactive protein and fibrinogen

To determine the threshold for identifying high-risk patients by PCSK9 levels, the Youden index was calculated across various PSCK9 values. The optimal cut-points were 50.4, 50.1 and 139.1 ng/mL in the whole cohort, DM patients and non-DM patients, respectively (Additional file 2: Fig. S1 and Table S3). Compared with the whole cohort and non-DM population, PCSK9 acquired the highest sensitivity (0.674), specificity (0.597) and overall accuracy (AUC: 0.634, Youden index: 0.271) for predicting MACE in DM patients. When stratified by the threshold, high PCSK9 levels (> 50.1 ng/mL) remained an independent predictor of MACEs in DM patients after multiple adjustment (HR: 3.344, 95% CI 1.160–6.897, P = 0.001) (Additional file 2: Table S4). The hazard increment associated with high levels of PCSK9 was still significant but attenuated in the whole cohort (> 50.4 ng/mL, HR: 1.616, 95% CI 1.148–2.273, P = 0.006) and non-DM patients (> 139.1 ng/mL, HR: 2.037, 95% CI 1.202–3.448, P = 0.008) when stratified by the optimal thresholds of each subgroup.

**Fig. 3:**
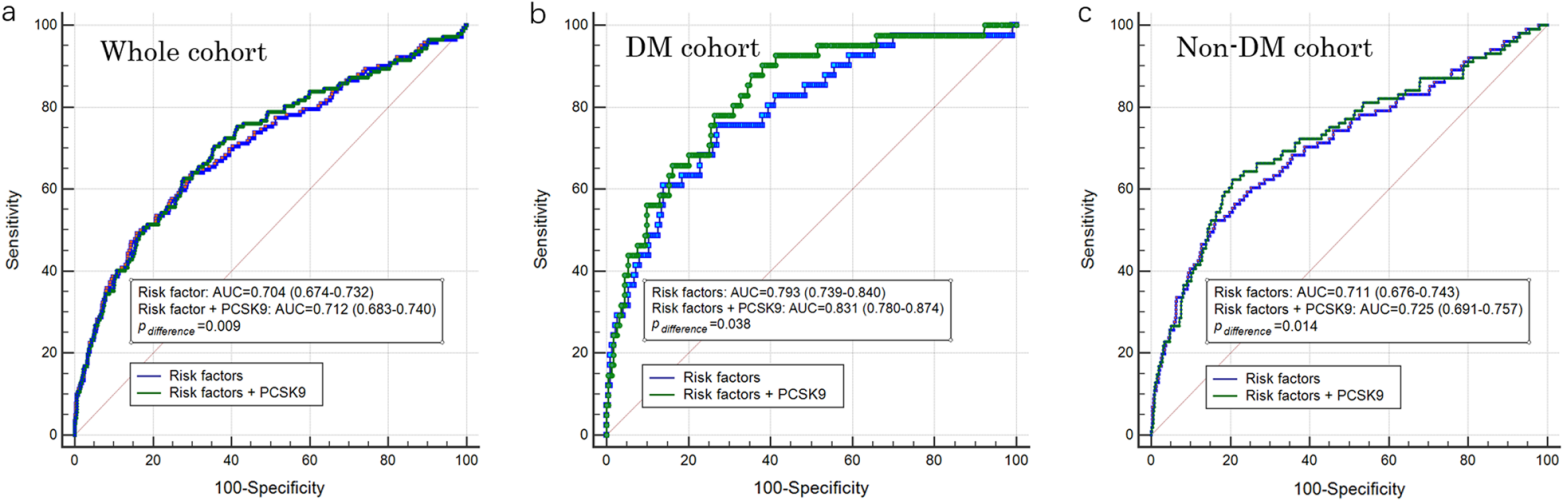
Receiver operating characteristic curves of risk models of established risk factors with or without levels of PCSK9 for predicting major adverse cardiovascular events in the whole cohort (**a**), DM patients (**b**) and non-DM patients (**c**). *PCSK9* proprotein convertase subtilisin/kexin type 9, *DM* diabetes mellitus, *Non-DM* non-diabetes mellitus, *AUC* area under the curve

To test the extra predictive value of PCSK9, we added PCSK9 levels to the models containing established risk factors for MACEs, including all variables with P < 0.10 in the univariable logistic regression (i.e., age, sex, body mass index, hypertension, smoking status, LVEF, TIMI score, coronary artery lesions, total cholesterol, HbA1c, leukocyte count, hemoglobin, platelet count, estimated glomerular filtration rate, hs-CRP and fibrinogen). ROC analysis showed that the AUC was significantly increased with the addition of PCSK9 levels in the whole cohort (AUC: 0.712 vs. 0.704, P _*difference*_ = 0.009). Similar improvements of AUC were also observed in subgroup analysis for DM patients (AUC: 0.831 vs. 0.793, P _*difference*_ = 0.038) and non-DM patients (AUC: 0.725 vs. 0.711, P _*difference*_ = 0.014) (Fig. 3). Taken together, the combination of PCSK9 levels and established risk factors further improved the risk prediction of long-term MACEs, especially for DM patients.

## Discussion

This study demonstrated that high circulating PCSK9 levels are independently correlated with an increased risk of MACEs in STEMI patients with DM undergoing primary PCI. The significant association of PCSK9 levels with hs-CRP, sP-selectin, sCD40L and platelet reactivity markers suggested that PCSK9 might contribute to the recurrence of adverse cardiovascular events, at least in part, through promoting inflammation and increasing platelet activation in DM patients during the acute stage of MI.

Recently, a positive association of PCSK9 levels with adverse cardiovascular events has been observed in general population, in patients with familial hypercholesterolemia, in stable CAD, in atrial fibrillation, and in those undergoing PCI [6, 14–17]. However, the prognostic value of PCSK9 for the risk of MACEs in ACS and/or DM patients remains undetermined. In a post-hoc analysis, Khoury et al. enrolled two DM cohorts (DIABHYCAR study and SURDIAGENE study) and found that PCSK9 levels were inconsistently associated with cardiovascular events in patients with DM [18]. A previous study including 2030 ACS patients (52.9% presenting with STEMI and 3% receiving coronary artery by-pass grafting) who admitted within 5 days of pain onset indicated that high initial PCSK9 levels did not predict all-cause death and MACEs at 1 year [7]. Another previous study recruited 1646 patients with acute MI (84.9% presenting with STEMI and only 47.5% undergoing reperfusion therapy) and showed that the baseline PCSK9 levels did not predict MACEs (including cardiac death, nonfatal MI, coronary revascularization, and ischemic stroke) within 1 year [9]. In contrast, a prospective study including 333 ACS patients (prior statin intake in nearly 30% of the participants) undergoing PCI indicated that the baseline PCSK9 levels were independently associated with increased ischemic cardiac outcomes at 1-year follow-up [8]. Similar to several previous studies [9, 10, 19, 20], the present study evaluated the baseline PCSK9 levels measured within 24 h of symptom onset just in STEMI patients undergoing successful primary PCI. Moreover, it has been well established that circulating PCSK9 levels could be increased by several commonly used lipid-lowering medicines such as statin, ezetimibe or berberine, and the duration of lipid-lowering treatment may also affect PCSK9 levels [21, 22]. Accordingly, in order to avoid these impacts on baseline PCSK9 levels, we excluded those who took any lipid-lowering medicines within 3 months before admission. The main results indicated that elevated PCSK9 levels were associated with a worse prognosis (2 years of median follow-up) in STEMI patients with DM even after adjustment for potential confounders. In addition, the combination of high PCSK9 levels with DM showed the greatest risk of MACEs, which is consistent with the results of a previous study enrolling stable CAD [11]. It should be noted that the differences with respect to study design (including participants, sample size, timing of blood samples collection, prior use of lipid-lowering medicines, and treatment regimen such as reperfusion therapy within the effective time window), selection of clinical outcomes and follow-up durations might all affect the results.

The existence of a close relationship between lipid and glucose metabolism has promoted the research of the possible participation of PCSK9 in glucose homeostasis. The present study indicated that PCSK9 levels were negatively associated with fasting glucose in DM patients, whereas no correlations were found in the whole cohort and non-DM patients. Moreover, consistent with several previous studies [9, 18, 23, 24], we did not find a significant association between baseline PCSK9 levels and HbA1C. Currently, it remains controversial regarding the relationships between circulating PCSK9 levels and glucose metabolic parameters, diabetic status and the risk of new onset diabetes, and further clarification is needed.

Our data showed that PCSK9 levels were not correlated with LDL-C and total cholesterol levels despite the lack of prior lipid-lowering therapy. Indeed, associations of PCSK9 levels with lipid metabolism-related parameters are inconsistent in different cardiovascular risk populations [6, 9, 11, 17, 18, 25]. Further, previous studies suggested that plasma PCSK9 levels were elevated at the acute phase of MI and were uncoupled from LDL-C levels which fell transiently following MI [20, 26, 27]. Recent studies indicated that PCSK9 could also predict cardiovascular events even in those patients despite statin therapy with well-controlled LDL-C levels [6], suggesting that the effects of PCSK9 on cardiovascular systems might be mediated by LDL-independent mechanisms. Several studies have shown the positive association between PCSK9 and triglycerides levels [6, 7, 9], and PCSK9 inhibition has been demonstrated to influence triglyceride-rich lipoprotein metabolism which is also a risk factor for cardiovascular disease and vascular inflammation [28, 29]. The present study also indicated that PCSK9 levels were positively correlated with triglycerides levels, and the correlation was stronger in DM patients than in non-DM patients. Additionally, we found that PCSK9 levels were negatively associated with HDL-C and positively associated with lipoprotein(a), although the strength of the associations was weak. As we know, dyslipidemia in DM is characterized mainly by high triglycerides levels, reduced HDL-C levels and normal or slightly increased LDL-C levels, which is different from non-DM populations. Therefore, further dedicated investigations are warranted to elucidate the relationship between circulating PCSK9 levels and lipid metabolism-related parameters along with the role of PCSK9 inhibition in the setting of ACS with or without DM.

Aside from lipid profiles, inflammation and platelet activation and reactivity also play key roles in the pathogenesis of recurrent ischemic events after ACS [30, 31]. The involvement of PCSK9 in systemic or vascular inflammation has been demonstrated by experimental studies [32–34]. Furthermore, clinical studies have also found a positive association between PCSK9 and hs-CRP levels [6, 7, 9, 17]. DM itself is associated with chronic low-level inflammation, so the association between PCSK9 and inflammation markers in DM may be different from non-DM populations. The present study indicated that high baseline PCSK9 levels were correlated with a higher degree of inflammation as measured by hs-CRP, and the correlation was stronger in DM patients than in non-DM patients. The association of PCSK9 with systemic inflammation might affect the prognostic value of PCSK9 for long-term outcomes. An intravascular ultrasound study by Cheng et al. also indicated a positive correlation of PCSK9 levels with absolute volume and fraction of necrotic core tissue of atherosclerotic plaques, which is responsible for coronary plaque inflammation [35]. Moreover, PCSK9 inhibition results in attenuated oxidized LDL-induced expression of proinflammatory chemokines, decreased macrophage and monocyte recruitment as well as necrotic core content in animal models [36]. However, whether PCSK9 inhibition represents a novel therapy for modulating inflammatory response in ACS patients with DM needs to further investigation.

Recent findings suggest a role of PCSK9 in the activation of thrombotic pathways. For example, PCSK9 knockout mice develop less arterial thrombosis and show reduced in vivo platelet activation upon arterial injury [37]. Moreover, recombinant human PCSK9 added in vitro to human platelets potentiated activation induced by weak agonists [37]. Importantly, in a clinical study including ACS patients undergoing PCI and receiving novel P2Y_12_ inhibitors (prasugrel or ticagrelor), a direct association between increased PCSK9 levels and high-on-treatment platelet reactivity was found [8]. Subsequently, a direct correlation between urinary excretion of 11-dehydro-thromboxane-B_2_, a marker of in vivo platelet activation, and circulating PCSK9 levels was reported in patients with atrial fibrillation [16]. The present study revealed that PCSK9 levels were positively associated with sP-selectin and sCD40L levels, two commonly used markers of platelet activation, and the correlations were stronger in DM patients than in non-DM patients. Additionally, we found that PCSK9 levels were significantly and positively associated with MPA and MA_ADP_ in DM patients treated with ticagrelor. However, no correlations between PCSK9 levels and platelet reactivity were found in non-DM patients treated with ticagrelor and in those treated with clopidogrel regardless of DM status. A more recent study by Ge et al. demonstrated that plasma PCSK9 directly enhances platelet activation and in vivo thrombosis by binding to platelet CD36 and thus activating the downstream signaling pathways, and PCSK9 inhibitors abolish the enhancing effects of PCSK9 [38]. In addition, Cammisotto et al. also found that high circulating levels of PCSK9 are associated with increased platelet activation with a mechanism involving CD36 and eventually Nox2 activation [39].

Although the clinical utility of PCSK9 levels to guide ACS treatment remains undetermined, the current study demonstrated that PCSK9 was potentially beneficial for early risk stratifications of STEMI patients, especially for diabetic patients, as the biomarker acquired the highest accuracy for predicting long-term ischemic events in the subgroup of DM. Moreover, PCSK9 significantly improved the accuracy of risk prediction for MACEs when combined with established clinical risk factors. These findings suggest that PCSK9 might offer additional prognostic insight beyond demographics, common comorbidities and hemodynamics, which might relate to its twofold mechanisms on both lipid lowering and inflammation/platelet pathways. It would be of clinical interest to further investigate whether PCSK9 measurements could identify patients who are potentially more suitable for more intensive lipid-lowering treatment (e.g., PCSK9 inhibitors, high-dose statins), anti-inflammatory and anti-thrombotic medications.

## Limitations

Although our findings provided additional information on the association between circulating PCSK9 levels and the risk of MACEs in STEMI patients, there were several limitations to be considered. Firstly, although the sample size was large enough, the incidence of several secondary endpoints was relatively low, which could lead to inadequate statistical power for subgroup analysis or assessment of individual outcomes. Secondly, we only measured the baseline PCSK9 concentration and did not evaluate the effects of its longitudinal change on MACEs during follow-up. Thirdly, as with the observational design of the study, only association but no cause-effect relationship could be determined. Fourthly, as samples were stored for 1 to 4 years prior to ELISA analysis, degradation of PCSK9 or unexpected changes may have occurred.

## Conclusions

The study showed that high circulating PCSK9 levels are independently associated with an increased risk of MACEs in STEMI patients with DM undergoing primary PCI. The significant correlation of PCSK9 with inflammation and platelet activation markers might contribute to the increased risk of adverse cardiovascular events in STEMI patients with high PCSK9 levels plus DM. The findings suggest a potential benefit of PCSK9 inhibition in the early phase of ACS, especially for patients with DM plus high PCSK9 levels, by a twofold mechanism on both lipid lowering and inflammation/platelet pathways.

## Supplementary Information

**Additional file 1. **ELISA Protocol.

**Additional file 2.** **Table S1.** Multivariable Cox regression analyses of PCSK9 levels for MACEs. **Table S2.** Multivariable Cox regression analyses of MACEs based on the combination of PCSK9 levels and diabetes status.  **Table S3.** Optimal cut-off threshold of PSCK9 for predicting MACEs in various patient population. **Table S4.** Associations of PCSK9 levels stratified by the optimal threshold with MACEs in various patient population. **Fig. S1. **Receiver operating characteristic curves of PCSK9 for predicting major adverse cardiovascular events in the whole cohort (**a**), DM patients (**b**) and non-DM patients (**c**).

## Data Availability

The datasets used and/or analyzed during the current study are available from the corresponding author on reasonable request.

## Abbreviations

ACS: Acute coronary syndrome
PCSK9: Proprotein convertase subtilisin/kexin type 9
LDL-C: Low-density lipoprotein cholesterol
MACEs: Major adverse cardiovascular events
CAD: Coronary artery disease
DM: Diabetes mellitus
STEMI: ST-segment elevation myocardial infarction
PCI: Percutaneous coronary intervention
sCD40L: Soluble CD40 ligand
sP-selectin: Soluble P-selectin
ELISA: Enzyme-linked immunosorbent assay
hs-CRP: High-sensitivity C reactive protein
LTA: Light transmittance aggregometry
TEG: Thrombelastography
ADP: Adenosine diphosphate
MPA: Maximal platelet aggregation
MA_ADP_: Maximum amplitude of ADP-induced platelet-fibrin clots
MI: Myocardial infarction
SD: Standard deviation
HR: Hazard ratios
CI: Confidence intervals
ROC: Receiver operating characteristic
AUC: Area under the curve
LVEF: Left ventricular ejection fraction
HDL-C: High-density lipoprotein cholesterol
