# Supplementary material for: Association of PCSK9 with inflammation and platelet activation markers and recurrent cardiovascular risks in STEMI patients undergoing primary PCI with or without diabetes
Source: Cardiovasc Diabetol. 2022 May 20;21:80. doi: 10.1186/s12933-022-01519-3 (PMC9123773; doi:10.1186/s12933-022-01519-3)
Supplement: Supplementary file 1 — Additional file 1. ELISA Protocol. [file 12933_2022_1519_MOESM1_ESM.docx]

**Additional file 1: ELISA Protocol**

**PCSK9**

| **Human Proprotein Convertase 9/PCSK9 Catalog Number: DY3888 (15 plates)** | |
| --- | --- |
| CALCULATION OF RESULTS | Average the duplicate readings for each standard, control, and sample and subtract the average zero standard optical density (O.D.). Create a standard curve by reducing the data using computer software capable of generating a four parameter logistic (4-PL) curve-fit. As an alternative, construct a standard curve by plotting the mean absorbance for each standard on the y-axis against the concentration on the x-axis and draw a best fit curve through the points on the graph. The data may be linearized by plotting the log of the human PCSK9 concentrations versus the log of the O.D. and the best fit line can be determined by regression analysis. This procedure will produce an adequate but less precise fit of the data. If samples have been diluted, the concentration read from the standard curve must be multiplied by the dilution factor. |
| TYPICAL DATA | This standard curve is only for demonstration purposes. A standard curve should be generated for each set of samples assayed. |
| SPECIFICITY | The following factors prepared at 50 ng/mL were assayed and exhibited no cross-reactivity or interference. Recombinant human: Furin LDL R Proprotein Convertase 1 Proprotein Convertase 7 Recombinant mouse: Proprotein Convertase 9 |
| TECHNICAL HINTS & LIMITATIONS | • We recommend the use of R&D Systems’ Reagent Diluent Concentrate 2 (Catalog # DY995) to prepare Reagent Diluent for use in this assay.  • The use of high quality Bovine Serum Albumin (BSA) for the Reagent Diluent is crucial for the optimum performance of the DuoSet® ELISA Development kit. Impurities such as proteases, binding proteins, soluble receptors or other interfering substances can be found to varying degrees in virtually all BSA preparations and can inhibit or interfere with the detection of certain analytes. If the standard curve appears suppressed, consider evaluating a different preparation of BSA.  • It is suggested to start Reagent Diluent optimization for serum and plasma samples by using PBS supplemented with 10-50% animal serum. Do not use buffers with animal serum to reconstitute or dilute the Detection Antibody or Streptavidin-HRP. • It is important that the Reagent Diluent selected for reconstitution and dilution of the standard reflects the environment of the samples being measured.  • Avoid microbial contamination of reagents and buffers.  • A thorough and consistent wash technique is essential for proper assay performance. Wash Buffer should be dispensed forcefully and removed completely from the wells by aspiration or decanting. Remove any remaining Wash Buffer by inverting the plate and blotting it against clean paper towels.  • Individual results may vary due to differences in technique, plasticware and water sources.  • It is recommended that all standards and samples be assayed in duplicate.  • The use of PBS from tablets may interfere in this assay. |
| TROUBLESHOOTING | Note: For more detailed troubleshooting, please visit: www.RnDSystems.com/ELISADevelopment  Poor Standard Curve • Impure BSA used for Reagent Diluent preparation. • Improper reconstitution and/or storage of standard. • Improper dilution of highest standard and standard curve. • Incomplete washing and/or aspiration of wells. • Unequal volumes added to wells/pipetting error. • Incorrect incubation times or temperatures. Poor Precision • Unequal volumes added to wells/pipetting error. • Incomplete washing and/or aspiration of wells. • Unequal mixing of reagents. Low or No color Development • Inadequate volume of substrate added to wells. • Incorrect incubation times or temperatures. • Impure BSA used for Reagent Diluent preparation. |
| INTENDED USE | For the development of sandwich ELISAs to measure natural and recombinant human Proprotein Convertase 9 (PCSK9). The Reagent Diluent recommended may be suitable for most cell culture supernate, serum, and plasma samples. The Reagent Diluent selected for use can alter the performance of an immunoassay. Reagent Diluent optimization for samples with complex matrices such as serum and plasma, may improve their performance in this assay. This kit contains sufficient materials to run ELISAs on at least fifteen 96 well plates, provided the following conditions are met: • The reagents are prepared as described in this package insert. • The assay is run as described in the General ELISA Protocol. • The recommended microplates, buffers, diluents, substrates, and solutions are used. |
| OTHER MATERIALS & SOLUTIONS REQUIRED DuoSet® | Ancillary Reagent Kit 2 (5 plates): (R&D Systems, Catalog # DY008) containing 96 well microplates, plate sealers, substrate solution, stop solution, plate coating buffer (PBS), wash buffer, and Reagent Diluent Concentrate 2. The components listed above may be purchased separately: 96 well microplates: (R&D Systems, Catalog # DY990). Plate Sealers: (R&D Systems, Catalog # DY992). PBS: 137 mM NaCl, 2.7 mM KCl, 8.1 mM Na2HPO4, 1.5 mM KH2PO4, pH 7.2-7.4, 0.2 μm filtered (R&D Systems, Catalog # DY006). Wash Buffer: 0.05% Tween® 20 in PBS, pH 7.2-7.4 (R&D Systems, Catalog # WA126). Reagent Diluent: 1% BSA in PBS, pH 7.2-7.4, 0.2 μm filtered (R&D Systems, Catalog # DY995). Quality of BSA is critical (see Technical Hints). Substrate Solution: 1:1 mixture of Color Reagent A (H2O2) and Color Reagent B (Tetramethylbenzidine) (R&D Systems, Catalog # DY999). Stop Solution: 2 N H2SO4 (R&D Systems, Catalog # DY994). |
| PRECAUTIONS | Some components in this kit contain a preservative which may cause an allergic skin reaction. Avoid breathing mist. The Stop Solution suggested for use with this kit is an acid solution. The Color Reagent B suggested for use with this kit may cause skin, eye, and respiratory irritation. Avoid breathing fumes. Wear protective gloves, clothing, eye, and face protection. Wash hands thoroughly after handling. Please refer to the MSDS on our website prior to use |
| CALIBRATION | This DuoSet® is calibrated against a highly purified NS0-expressed recombinant human PCSK9 produced at R&D Systems. |
| REAGENT PREPARATION | Bring all reagents to room temperature before use. Allow all components to sit for a minimum of 15 minutes with gentle agitation after initial reconstitution. Working dilutions should be prepared and used immediately. Streptavidin-HRP: 1.0 mL of streptavidin conjugated to horseradishperoxidase. Dilute to the working concentration specified on the vial label using Reagent Diluent. Rat Anti-Human PCSK9 Capture Antibody: Refer to the lot-specific C of A for amount supplied. Reconstitute with 1.0 mL of PBS. Dilute in PBS without carrier protein to the working concentration indicated on the C of A. Biotinylated Sheep Anti-Human PCSK9 Detection Antibody: Refer to the lot-specific C of A for amount supplied. Reconstitute with 1.0 mL of Reagent Diluent. Dilute in Reagent Diluent to the working concentration indicated on the C of A. Recombinant Human PCSK9 Standard: Refer to the lot-specific C of A for amount supplied. Reconstitute each vial with 0.5 mL of Reagent Diluent. A seven point standard curve using 2-fold serial dilutions in Reagent Diluent is recommended. Prepare 1000 μL of high standard per plate assayed at the concentration indicated on the C of A |
| GENERAL ELISA PROTOCOL | Plate Preparation 1. Dilute the Capture Antibody to the working concentration in PBS without carrier protein. Immediately coat a 96-well microplate with 100 μL per well of the diluted Capture Antibody. Seal the plate and incubate overnight at room temperature. 2. Aspirate each well and wash with Wash Buffer, repeating the process two times for a total of three washes. Wash by filling each well with Wash Buffer (400 μL) using a squirt bottle, manifold dispenser, or autowasher. Complete removal of liquid at each step is essential for good performance. After the last wash, remove any remaining Wash Buffer by aspirating or by inverting the plate and blotting it against clean paper towels. 3. Block plates by adding 300 μL of Reagent Diluent to each well. Incubate at room temperature for a minimum of 1 hour. 4. Repeat the aspiration/wash as in step 2. The plates are now ready for sample addition. Assay Procedure 1. Add 100 μL of sample or standards in Reagent Diluent, or an appropriate diluent, per well. Cover with an adhesive strip and incubate 2 hours at room temperature. 2. Repeat the aspiration/wash as in step 2 of Plate Preparation. 3. Add 100 μL of the Detection Antibody, diluted in Reagent Diluent, to each well. Cover with a new adhesive strip and incubate 2 hours at room temperature. 4. Repeat the aspiration/wash as in step 2 of Plate Preparation. 5. Add 100 μL of the working dilution of Streptavidin-HRP to each well. Cover the plate and incubate for 20 minutes at room temperature. Avoid placing the plate in direct light. 6. Repeat the aspiration/wash as in step 2. 7. Add 100 μL of Substrate Solution to each well. Incubate for 20 minutes at room temperature. Avoid placing the plate in direct light. 8. Add 50 μL of Stop Solution to each well. Gently tap the plate to ensure thorough mixing. 9. Determine the optical density of each well immediately, using a microplate reader set to 450 nm. If wavelength correction is available, set to 540 nm or 570 nm. If wavelength correction is not available, subtract readings at 540 nm or 570 nm from the readings at 450 nm. This subtraction will correct for optical imperfections in the plate. Readings made directly at 450 nm without correction may be higher and less accurate. |

**sCD40L**

| **Human CD40 Ligand/TNFSF5 Catalog Number: DY617 (15 plates)** | |
| --- | --- |
| CALCULATION OF RESULTS | Average the duplicate readings for each standard, control, and sample and subtract the average zero standard optical density (O.D.). Create a standard curve by reducing the data using computer software capable of generating a four parameter logistic (4-PL) curve-fit. As an alternative, construct a standard curve by plotting the mean absorbance for each standard on the y-axis against the concentration on the x-axis and draw a best fit curve through the points on the graph. The data may be linearized by plotting the log of the human CD40 Ligand concentrations versus the log of the O.D. and the best fit line can be determined by regression analysis. This procedure will produce an adequate but less precise fit of the data. If samples have been diluted, the concentration read from the standard curve must be multiplied by the dilution factor. |
| TYPICAL DATA | This standard curve is only for demonstration purposes. A standard curve should be generated for each set of samples assayed. |
| SPECIFICITY | The following factors prepared at 50 ng/mL were assayed and exhibited no cross-reactivity or interference. |
| TECHNICAL HINTS & LIMITATIONS | • We recommend the use of R&D Systems’ Reagent Diluent Concentrate 2 (Catalog # DY995) to prepare Reagent Diluent for use in this assay.  • The use of high quality Bovine Serum Albumin (BSA) for the Reagent Diluent is crucial for the optimum performance of the DuoSet ELISA Development kit. Impurities such as proteases, binding proteins, soluble receptors or other interfering substances can be found to varying degrees in virtually all BSA preparations and can inhibit or interfere with the detection of certain analytes. If the standard curve appears suppressed, consider evaluating a different preparation of BSA.  • It is suggested to start Reagent Diluent optimization for serum and plasma samples by using PBS supplemented with 10-50% animal serum. Do not use buffers with animal serum to reconstitute or dilute the Detection Antibody or Streptavidin-HRP.  • It is important that the Reagent Diluent selected for reconstitution and dilution of the standard reflects the environment of the samples being measured.  • Avoid microbial contamination of reagents and buffers.  • A thorough and consistent wash technique is essential for proper assay performance. Wash Buffer should be dispensed forcefully and removed completely from the wells by aspiration or decanting. Remove any remaining Wash Buffer by inverting the plate and blotting it against clean paper towels.  • Individual results may vary due to differences in technique, plasticware and water sources.  • It is recommended that all standards and samples be assayed in duplicate.  • The use of PBS from tablets may interfere in this assay. |
| INTENDED USE | For the development of sandwich ELISAs to measure natural and recombinant human CD40 Ligand. The Reagent Diluent recommended may be suitable for most cell culture supernate, serum, and plasma samples. The Reagent Diluent selected for use can alter the performance of an immunoassay. Reagent Diluent optimization for samples with complex matrices such as serum and plasma, may improve their performance in this assay. This kit contains sufficient materials to run ELISAs on at least fifteen 96 well plates, provided the following conditions are met: • The reagents are prepared as described in this package insert. • The assay is run as described in the General ELISA Protocol. • The recommended microplates, buffers, diluents, substrates, and solutions are used. |
| OTHER MATERIALS & SOLUTIONS REQUIRED DuoSet® | DuoSet Ancillary Reagent Kit 2 (5 plates): (R&D Systems, Catalog # DY008) containing 96 well microplates, plate sealers, substrate solution, stop solution, plate coating buffer (PBS), wash buffer, and Reagent Diluent Concentrate 2. The components listed above may be purchased separately: 96 well microplates: (R&D Systems, Catalog # DY990). Plate Sealers: (R&D Systems, Catalog # DY992). PBS: 137 mM NaCl, 2.7 mM KCl, 8.1 mM Na2HPO4, 1.5 mM KH2PO4, pH 7.2-7.4, 0.2 μm filtered (R&D Systems, Catalog # DY006). Wash Buffer: 0.05% Tween® 20 in PBS, pH 7.2-7.4 (R&D Systems, Catalog # WA126). Reagent Diluent: 1% BSA in PBS, pH 7.2-7.4, 0.2 μm filtered (R&D Systems, Catalog # DY995). Quality of BSA is critical (see Technical Hints). Substrate Solution: 1:1 mixture of Color Reagent A (H2O2) and Color Reagent B (Tetramethylbenzidine) (R&D Systems, Catalog # DY999). Stop Solution: 2 N H2SO4 (R&D Systems, Catalog # DY994). |
| PRECAUTIONS | Some components in this kit contain ProClin® which may cause an allergic skin reaction. Avoid breathing mist. The Stop Solution suggested for use with this kit is an acid solution. The Color Reagent B suggested for use with this kit may cause skin, eye, and respiratory irritation. Avoid breathing fumes. Wear protective gloves, clothing, eye, and face protection. Wash hands thoroughly after handling. Please refer to the MSDS on our website prior to use. |
| CALIBRATION | This DuoSet is calibrated against a highly purified E. coli-expressed recombinant human CD40 Ligand produced at R&D Systems. |
| REAGENT PREPARATION | Bring all reagents to room temperature before use. Allow all components to sit for a minimum of 15 minutes with gentle agitation after initial reconstitution. Working dilutions should be prepared and used immediately. Streptavidin-HRP: 1.0 mL of streptavidin conjugated to horseradishperoxidase. Dilute to the working concentration specified on the vial label using Reagent Diluent. Goat Anti-Human CD40 Ligand Capture Antibody: Refer to the lot-specific C of A for amount supplied. Reconstitute with 1.0 mL of PBS. Dilute in PBS without carrier protein to the working concentration indicated on the C of A. Biotinylated Goat Anti-Human CD40 Ligand Detection Antibody: Refer to the lot-specific C of A for amount supplied. Reconstitute with 1.0 mL of Reagent Diluent. Dilute in Reagent Diluent to the working concentration indicated on the C of A. Recombinant Human CD40 Ligand Standard: Refer to the lot-specific C of A for amount supplied. Reconstitute each vial with 0.5 mL of Reagent Diluent. A seven point standard curve using 2-fold serial dilutions in Reagent Diluent is recommended. Prepare 1000 μL of high standard per plate assayed at the concentration indicated on the C of A. |
| GENERAL ELISA PROTOCOL | Plate Preparation  1. Dilute the Capture Antibody to the working concentration in PBS without carrier protein. Immediately coat a 96-well microplate with 100 μL per well of the diluted Capture Antibody. Seal the plate and incubate overnight at room temperature. 2. Aspirate each well and wash with Wash Buffer, repeating the process two times for a total of three washes. Wash by filling each well with Wash Buffer (400 μL) using a squirt bottle, manifold dispenser, or autowasher. Complete removal of liquid at each step is essential for good performance. After the last wash, remove any remaining Wash Buffer by aspirating or by inverting the plate and blotting it against clean paper towels. 3. Block plates by adding 300 μL of Reagent Diluent to each well. Incubate at room temperature for a minimum of 1 hour. 4. Repeat the aspiration/wash as in step 2. The plates are now ready for sample addition.  Assay Procedure  1. Add 100 μL of sample or standards in Reagent Diluent, or an appropriate diluent, per well. Cover with an adhesive strip and incubate 2 hours at room temperature. 2. Repeat the aspiration/wash as in step 2 of Plate Preparation. 3. Add 100 μL of the Detection Antibody, diluted in Reagent Diluent, to each well. Cover with a new adhesive strip and incubate 2 hours at room temperature. 4. Repeat the aspiration/wash as in step 2 of Plate Preparation. 5. Add 100 μL of the working dilution of Streptavidin-HRP to each well. Cover the plate and incubate for 20 minutes at room temperature. Avoid placing the plate in direct light. 6. Repeat the aspiration/wash as in step 2. 7. Add 100 μL of Substrate Solution to each well. Incubate for 20 minutes at room temperature. Avoid placing the plate in direct light. 8. Add 50 μL of Stop Solution to each well. Gently tap the plate to ensure thorough mixing. 9. Determine the optical density of each well immediately, using a microplate reader set to 450 nm. If wavelength correction is available, set to 540 nm or 570 nm. If wavelength correction is not available, subtract readings at 540 nm or 570 nm from the readings at 450 nm. This subtraction will correct for optical imperfections in the plate. Readings made directly at 450 nm without correction may be higher and less accurate. |

**sP-selectin**

| **Human P-Selectin/CD62P Catalog Number: DY137 (15 plates)** | |
| --- | --- |
| CALCULATION OF RESULTS | Average the duplicate readings for each standard, control, and sample and subtract the average zero standard optical density (O.D.). Create a standard curve by reducing the data using computer software capable of generating a four parameter logistic (4-PL) curve-fit. As an alternative, construct a standard curve by plotting the mean absorbance for each standard on the y-axis against the concentration on the x-axis and draw a best fit curve through the points on the graph. The data may be linearized by plotting the log of the human P-Selectin concentrations versus the log of the O.D. and the best fit line can be determined by regression analysis. This procedure will produce an adequate but less precise fit of the data. If samples have been diluted, the concentration read from the standard curve must be multiplied by the dilution factor. |
| TYPICAL DATA | This standard curve is only for demonstration purposes. A standard curve should be generated for each set of samples assayed. |
| SPECIFICITY | The following factors prepared at 50 ng/mL were assayed and exhibited no cross-reactivity or interference. |
| TECHNICAL HINTS & LIMITATIONS | • We recommend the use of R&D Systems’ Reagent Diluent Concentrate 2 (Catalog # DY995) to prepare Reagent Diluent for use in this assay.  • The use of high quality Bovine Serum Albumin (BSA) for the Reagent Diluent is crucial for the optimum performance of the DuoSet ELISA Development kit. Impurities such as proteases, binding proteins, soluble receptors or other interfering substances can be found to varying degrees in virtually all BSA preparations and can inhibit or interfere with the detection of certain analytes. If the standard curve appears suppressed, consider evaluating a different preparation of BSA.  • It is suggested to start Reagent Diluent optimization for serum and plasma samples by using PBS supplemented with 10-50% animal serum. Do not use buffers with animal serum to reconstitute or dilute the Detection Antibody or Streptavidin-HRP.  • It is important that the Reagent Diluent selected for reconstitution and dilution of the standard reflects the environment of the samples being measured.  • Avoid microbial contamination of reagents and buffers.  • A thorough and consistent wash technique is essential for proper assay performance. Wash Buffer should be dispensed forcefully and removed completely from the wells by aspiration or decanting. Remove any remaining Wash Buffer by inverting the plate and blotting it against clean paper towels.  • Individual results may vary due to differences in technique, plasticware and water sources.  • It is recommended that all standards and samples be assayed in duplicate.  • The use of PBS from tablets may interfere in this assay. |
| INTENDED USE | For the development of sandwich ELISAs to measure natural and recombinant human P-Selectin. The Reagent Diluent recommended may be suitable for most cell culture supernate, serum, and plasma samples. The Reagent Diluent selected for use can alter the performance of an immunoassay. Reagent Diluent optimization for samples with complex matrices such as serum and plasma, may improve their performance in this assay. This kit contains sufficient materials to run ELISAs on at least fifteen 96 well plates, provided the following conditions are met: • The reagents are prepared as described in this package insert. • The assay is run as described in the General ELISA Protocol. • The recommended microplates, buffers, diluents, substrates, and solutions are used. |
| OTHER MATERIALS & SOLUTIONS REQUIRED DuoSet® | DuoSet Ancillary Reagent Kit 2 (5 plates): (R&D Systems, Catalog # DY008) containing 96 well microplates, plate sealers, substrate solution, stop solution, plate coating buff er (PBS), wash buff er, and Reagent Diluent Concentrate 2. The components listed above may be purchased separately: 96 well microplates: (R&D Systems, Catalog # DY990). Plate Sealers: (R&D Systems, Catalog # DY992). PBS: 137 mM NaCl, 2.7 mM KCl, 8.1 mM Na2HPO4, 1.5 mM KH2PO4, pH 7.2-7.4, 0.2 μm fi ltered (R&D Systems, Catalog # DY006). Wash Buff er: 0.05% Tween® 20 in PBS, pH 7.2-7.4 (R&D Systems, Catalog # WA126). Reagent Diluent: 1% BSA in PBS, pH 7.2-7.4, 0.2 μm fi ltered (R&D Systems, Catalog # DY995). Quality of BSA is critical (see Technical Hints). Substrate Solution: 1:1 mixture of Color Reagent A (H2O2) and Color Reagent B (Tetramethylbenzidine) (R&D Systems, Catalog # DY999). Stop Solution: 2 N H2SO4 (R&D Systems, Catalog # DY994). |
| PRECAUTIONS | Some components in this kit contain ProClin® which may cause an allergic skin reaction. Avoid breathing mist. The Stop Solution suggested for use with this kit is an acid solution. The Color Reagent B suggested for use with this kit may cause skin, eye, and respiratory irritation. Avoid breathing fumes. Wear protective gloves, clothing, eye, and face protection. Wash hands thoroughly after handling. Please refer to the MSDS on our website prior to use. |
| CALIBRATION | This DuoSet is calibrated against a highly purifi ed CHO cell-expressed recombinant human P-Selectin produced at R&D Systems. |
| REAGENT PREPARATION | Bring all reagents to room temperature before use. Allow all components to sit for a minimum of 15 minutes with gentle agitation after initial reconstitution. Working dilutions should be prepared and used immediately. Streptavidin-HRP: 1.0 mL of streptavidin conjugated to horseradishperoxidase. Dilute to the working concentration specifi ed on the vial label using Reagent Diluent. Mouse Anti-Human P-Selectin Capture Antibody: Refer to the lot-specifi c C of A for amount supplied. Reconstitute with 1.0 mL of PBS. Dilute in PBS without carrier protein to the working concentration indicated on the C of A. Biotinylated Sheep Anti-Human P-Selectin Detection Antibody: Refer to the lot-specifi c C of A for amount supplied. Reconstitute with 1.0 mL of Reagent Diluent. Dilute in Reagent Diluent to the working concentration indicated on the C of A. Recombinant Human P-Selectin Standard: Refer to the lot-specifi c C of A for amount supplied. Reconstitute each vial with 0.5 mL of Reagent Diluent. A seven point standard curve using 2-fold serial dilutions in Reagent Diluent is recommended. Prepare 1000 μL of high standard per plate assayed at the concentration indicated on the C of A. |
| GENERAL ELISA PROTOCOL | Plate Preparation  1.Dilute the Capture Antibody to the working concentration in PBS without carrier protein. Immediately coat a 96-well microplate with 100 μL per well of the diluted Capture Antibody. Seal the plate and incubate overnight at room temperature. 2. Aspirate each well and wash with Wash Buff er, repeating the process two times for a total of three washes. Wash by fi lling each well with Wash Buff er (400 μL) using a squirt bottle, manifold dispenser, or autowasher. Complete removal of liquid at each step is essential for good performance. After the last wash, remove any remaining Wash Buff er by aspirating or by inverting the plate and blotting it against clean paper towels. 3. Block plates by adding 300 μL of Reagent Diluent to each well. Incubate at room temperature for a minimum of 1 hour. 4. Repeat the aspiration/wash as in step 2. The plates are now ready for sample addition.  Assay Procedure 1. Add 100 μL of sample or standards in Reagent Diluent, or an appropriate diluent, per well. Cover with an adhesive strip and incubate 2 hours at room temperature. 2. Repeat the aspiration/wash as in step 2 of Plate Preparation. 3. Add 100 μL of the Detection Antibody, diluted in Reagent Diluent, to each well. Cover with a new adhesive strip and incubate 2 hours at room temperature. 4. Repeat the aspiration/wash as in step 2 of Plate Preparation. 5. Add 100 μL of the working dilution of Streptavidin-HRP to each well. Cover the plate and incubate for 20 minutes at room temperature. Avoid placing the plate in direct light. 6. Repeat the aspiration/wash as in step 2. 7. Add 100 μL of Substrate Solution to each well. Incubate for 20 minutes at room temperature. Avoid placing the plate in direct light. 8. Add 50 μL of Stop Solution to each well. Gently tap the plate to ensure thorough mixing. 9. Determine the optical density of each well immediately, using a microplate reader set to 450 nm. If wavelength correction is available, set to 540 nm or 570 nm. If wavelength correction is not available, subtract readings at 540 nm or 570 nm from the readings at 450 nm. This subtraction will correct for optical imperfections in the plate. Readings made directly at 450 nm without correction may be higher and less accurate. |
