# Supplementary material for: Association of PCSK9 with inflammation and platelet activation markers and recurrent cardiovascular risks in STEMI patients undergoing primary PCI with or without diabetes
Source: Cardiovasc Diabetol. 2022 May 20;21:80. doi: 10.1186/s12933-022-01519-3 (PMC9123773; doi:10.1186/s12933-022-01519-3)
Supplement: Supplementary file 2 — Additional file 2. Table S1. Multivariable Cox regression analyses of PCSK9 levels for MACEs. Table S2. Multivariable Cox regression analyses of MACEs based on the combination of PCSK9 levels and diabetes status. Table S3. Optimal cut-off threshold of PSCK9 for predicting MACEs in various patient population. Table S4. Associations of PCSK9 levels stratified by the optimal threshold with MACEs in various patient population. Fig. S1. Receiver operating characteristic curves of PCSK9 for predicting major adverse cardiovascular events in the whole cohort (a), DM patients (b) and non-DM patients (c). [file 12933_2022_1519_MOESM2_ESM.docx]

**Additional file 2: Table S1** **Multivariable Cox regression analyses of PCSK9 levels for MACEs**

| **Variables** | **Univariate** | |  | **Multivariate** | |
| --- | --- | --- | --- | --- | --- |
|  | **HR (95% CI)** | **P value** |  | **HR (95% CI)** | **P value** |
| Whole cohort |  |  |  |  |  |
| Age | 1.033 (1.019-1.046) | < 0.001 |  |  |  |
| Male | 0.643 (0.447-0.924) | 0.017 |  |  |  |
| Body mass index | 0.958 (0.916-1.002) | 0.059 |  |  |  |
| Hypertension | 1.623 (1.155-2.282) | 0.005 |  |  |  |
| Smoking | 0.678 (0.493-0.932) | 0.017 |  |  |  |
| LVEF | 0.944 (0.930-0.959) | < 0.001 |  | 0.947 (0.930-0.966) | < 0.001 |
| TIMI score | 1.260 (1.180-1.346) | < 0.001 |  |  |  |
| Coronary artery lesions |  |  |  |  |  |
| DVD vs. SVD | 1.880 (1.238-2.857) | 0.003 |  | 1.706 (1.086-2.681) | 0.020 |
| TVD vs. SVD | 0.811 (0.565-1.165) | 0.257 |  |  |  |
| Total cholesterol | 0.862 (0.731-1.016) | 0.077 |  |  |  |
| HbA1c | 1.093 (0.998-1.196) | 0.055 |  |  |  |
| Leukocyte count | 1.052 (1.005-1.102) | 0.030 |  |  |  |
| Hemoglobin | 0.988 (0.979-0.997) | 0.007 |  |  |  |
| eGFR | 0.985 (0.980-0.991) | < 0.001 |  |  |  |
| hs-CRP | 1.058 (1.020-1.097) | 0.003 |  |  |  |
| Fibrinogen | 1.044 (1.009-1.081) | 0.013 |  |  |  |
| PCSK9, ng/mL  > 43.5 vs. ≤ 43.5 | 1.420 (1.033-1.953) | 0.031 |  | 1.361 (0.964-1.923) | 0.080 |
| DM |  |  |  |  |  |
| Age | 1.044 (1.017-1.072) | 0.001 |  |  |  |
| Male | 1.624 (0.892-2.957) | 0.112 |  |  |  |
| Body mass index | 0.985 (0.912-1.064) | 0.704 |  |  |  |
| Hypertension | 2.926 (1.309-6.543) | 0.009 |  | 2.743 (1.091-6.875) | 0.032 |
| Smoking | 0.499 (0.153-0.935) | 0.030 |  |  |  |
| LVEF | 0.939 (0.906-0.974) | 0.001 |  | 0.938 (0.899-0.978) | 0.003 |
| TIMI score | 1.272 (1.137-1.423) | < 0.001 |  |  |  |
| Coronary artery lesions |  |  |  |  |  |
| DVD vs. SVD | 1.241 (0.663-2.325) | 0.500 |  |  |  |
| TVD vs. SVD | 1.957 (0.797-4.808) | 0.143 |  |  |  |
| Total cholesterol | 0.999 (0.992-1.007) | 0.892 |  |  |  |
| HbA1c | 1.238 (1.055-1.453) | 0.009 |  |  |  |
| Leukocyte count | 1.036 (0.938-1.146) | 0.484 |  |  |  |
| Hemoglobin | 0.984 (0.968-0.999) | 0.041 |  |  |  |
| eGFR | 0.976 (0.965-0.988) | < 0.001 |  |  |  |
| hs-CRP | 1.036 (0.970-1.106) | 0.289 |  |  |  |
| Fibrinogen | 1.107 (0.946-1.296) | 0.204 |  |  |  |
| PCSK9, ng/mL  > 43.5 vs. ≤ 43.5 | 2.200 (1.195-4.048) | 0.011 |  | 2.283 (1.094-4.764) | 0.028 |
| Non-DM |  |  |  |  |  |
| Age | 1.028 (1.013-1.044) | < 0.001 |  |  |  |
| Male | 0.670 (0.420-1.069) | 0.093 |  |  |  |
| Body mass index | 0.945 (0.896-0.997) | 0.039 |  |  |  |
| Hypertension | 1.353 (0.918-1.994) | 0.126 |  |  |  |
| Smoking | 0.763 (0.524-1.112) | 0.160 |  |  |  |
| LVEF | 0.945 (0.929-0.961) | < 0.001 |  | 0.958 (0.937-0.979) | < 0.001 |
| TIMI score | 1.253 (1.154-1.361) | < 0.001 |  |  |  |
| Coronary artery lesions |  |  |  |  |  |
| DVD vs. SVD | 1.880 (1.168-3.030) | 0.009 |  | 0.569 (0.964-0.336) | 0.036 |
| TVD vs. SVD | 1.479 (0.948-2.304) | 0.084 |  |  |  |
| Total cholesterol | 0.995 (0.990-1.000) | 0.049 |  |  |  |
| HbA1c | 0.982 (0.814-1.184) | 0.850 |  |  |  |
| Leukocyte count | 1.060 (1.006-1.117) | 0.028 |  |  |  |
| Hemoglobin | 0.990 (0.980-1.001) | 0.062 |  |  |  |
| eGFR | 0.988 (0.981-0.994) | < 0.001 |  |  |  |
| hs-CRP | 1.067 (1.021-1.115) | 0.004 |  |  |  |
| Fibrinogen | 1.041 (1.002-1.081) | 0.037 |  |  |  |
| PCSK9, ng/mL  > 43.5 vs. ≤ 43.5 | 1.207 (0.828-1.759) | 0.329 |  | 1.145 (0.764-1.718) | 0.512 |

*PCSK9* proprotein convertase subtilisin/kexin type 9, *MACEs* major adverse cardiac events (including all-cause death, recurrent myocardial infarction, ischemic stroke and rehospitalization for heart failure), *HR* hazard ratio, *CI* confidence interval, *LVEF* left ventricular ejection fraction, *TIMI* thrombolysis in myocardial infarction, *DVD* double vessel disease, *SVD* single vessel disease, *TVD* triple vessel disease, *HbA1C* hemoglobin A1c, *eGFR* estimated glomerular filtration rate, *hs-CRP* high-sensitivity C reactive protein, *DM* diabetes mellitus, *Non-DM* non-diabetes mellitus.

**Additional file 2: Table S2 Multivariable Cox regression analyses of MACEs based on the combination of PCSK9 levels and diabetes status**

| **Variables** | **Univariate** | |  | **Multivariate** | |
| --- | --- | --- | --- | --- | --- |
|  | **HR (95% CI)** | **P value** |  | **HR (95% CI)** | **P value** |
| Age | 1.033 (1.019-1.046) | < 0.001 |  |  |  |
| Male | 0.643 (0.447-0.924) | 0.017 |  |  |  |
| Body mass index | 0.958 (0.916-1.002) | 0.059 |  |  |  |
| Hypertension | 1.623 (1.155-2.282) | 0.005 |  |  |  |
| Smoking | 0.678 (0.493-0.932) | 0.017 |  |  |  |
| LVEF | 0.944 (0.930-0.959) | < 0.001 |  | 0.949 (0.932-0.967) | < 0.001 |
| TIMI score | 1.260 (1.180-1.346) | < 0.001 |  |  |  |
| Coronary artery lesions |  |  |  |  |  |
| DVD vs. SVD | 1.880 (1.238-2.857) | 0.003 |  | 1.838 (1.170-2.890) | 0.008 |
| TVD vs. SVD | 0.811 (0.565-1.165) | 0.257 |  |  |  |
| Total cholesterol | 0.862 (0.731-1.016) | 0.077 |  |  |  |
| HbA1c | 1.093 (0.998-1.196) | 0.055 |  |  |  |
| Leukocyte count | 1.052 (1.005-1.102) | 0.030 |  |  |  |
| Hemoglobin | 0.988 (0.979-0.997) | 0.007 |  |  |  |
| eGFR | 0.985 (0.980-0.991) | < 0.001 |  |  |  |
| hs-CRP | 1.058 (1.020-1.097) | 0.003 |  |  |  |
| Fibrinogen | 1.044 (1.009-1.081) | 0.013 |  |  |  |
| PCSK9, ng/mL |  |  |  |  |  |
| PCSK9>43.5+Non-DM vs. PCSK9≤43.5+Non-DM | 1.757 (1.119-2.762) | 0.014 |  | 1.256 (0.708-2.227) | 0.436 |
| PCSK9≤43.5 + DM vs. PCSK9≤43.5+Non-DM | 1.468 (0.945-2.283) | 0.087 |  | 1.045 (0.603-1.812) | 0.874 |
| PCSK9>43.5+DM vs. PCSK9≤43.5+Non-DM | 2.188 (1.190-4.016) | 0.012 |  | 1.996 (1.047-3.817) | 0.036 |

*MACEs* major adverse cardiac events (including all-cause death, recurrent myocardial infarction, ischemic stroke and rehospitalization for heart failure), *PCSK9* proprotein convertase subtilisin/kexin type 9, *HR* hazard ratio, *CI* confidence interval, *LVEF* left ventricular ejection fraction, *TIMI* thrombolysis in myocardial infarction, *DVD* double vessel disease, *SVD* single vessel disease, *TVD* triple vessel disease, *HbA1C* hemoglobin A1c, *eGFR* estimated glomerular filtration rate, *hs-CRP* high-sensitivity C reactive protein, *Non-DM* non-diabetes mellitus, *DM* diabetes mellitus.

**Additional file 2: Table S3 Optimal cut-off threshold of PSCK9 for predicting MACEs in various patient population**

| **Population** | **AUC (95% CI)** | **Threshold (ng/mL)** | **Sensitivity** | **Specificity** | **Youden index** |
| --- | --- | --- | --- | --- | --- |
| **Whole cohort** | 0.558 (0.507-0.609) | 50.4 | 0.548 | 0.580 | 0.129 |
| **DM cohort** | 0.634 (0.549-0.719) | 50.1 | 0.674 | 0.597 | 0.271 |
| **Non-DM cohort** | 0.527 (0.464-0.589) | 139.1 | 0.183 | 0.910 | 0.093 |

*PCSK9* proprotein convertase subtilisin/kexin type 9, *MACEs* major adverse cardiac events (including all-cause death, recurrent myocardial infarction, ischemic stroke and rehospitalization for heart failure), *AUC* Area under the curve, *DM* diabetes mellitus, *Non-DM* non-diabetes mellitus.

**Additional file 2: Table S4 Associations of PCSK9 levels stratified by the optimal threshold with MACEs in various patient population**

| **Optimal threshold** | **MACEs event rate** | | **Unadjusted**  **HR (95% CI)** | **P value** | **Adjusted***  **HR (95% CI)** | **P value** |
| --- | --- | --- | --- | --- | --- | --- |
|  | **Low PCSK9** | **High PCSK9** |  |  |  |  |
| **Whole cohort** | | | | | | |
| 50.4 ng/mL | 70/576 (12.2) | 85/451 (18.8) | 1.677 (1.222-2.303) | 0.001 | 1.616 (1.148-2.273) | 0.006 |
| **DM cohort** | | | | | | |
| 50.1 ng/mL | 15/153 (9.8) | 31/124 (25.0) | 3.236 (1.742-6.024) | <0.001 | 3.344 (1.610-6.897) | 0.001 |
| **Non-DM cohort** | | | | | | |
| 139.1 ng/mL | 89/672 (13.2) | 20/78 (25.6) | 1.873 (1.151-3.040) | 0.012 | 2.037 (1.202-3.448) | 0.008 |

*PCSK9* proprotein convertase subtilisin/kexin type 9, *MACEs* major adverse cardiac events (including all-cause death, recurrent myocardial infarction, ischemic stroke and rehospitalization for heart failure), *HR* hazard ratio, *CI* confidence interval, *DM* diabetes mellitus, *Non-DM* non-diabetes mellitus. *Adjusted for age, sex, body mass index, hypertension, smoking status, left ventricular ejection fraction, thrombolysis in myocardial infarction score, coronary artery lesions, total cholesterol, HbA1c, leukocyte count, hemoglobin, estimated glomerular filtration rate, high-sensitivity C reactive protein and fibrinogen.

**
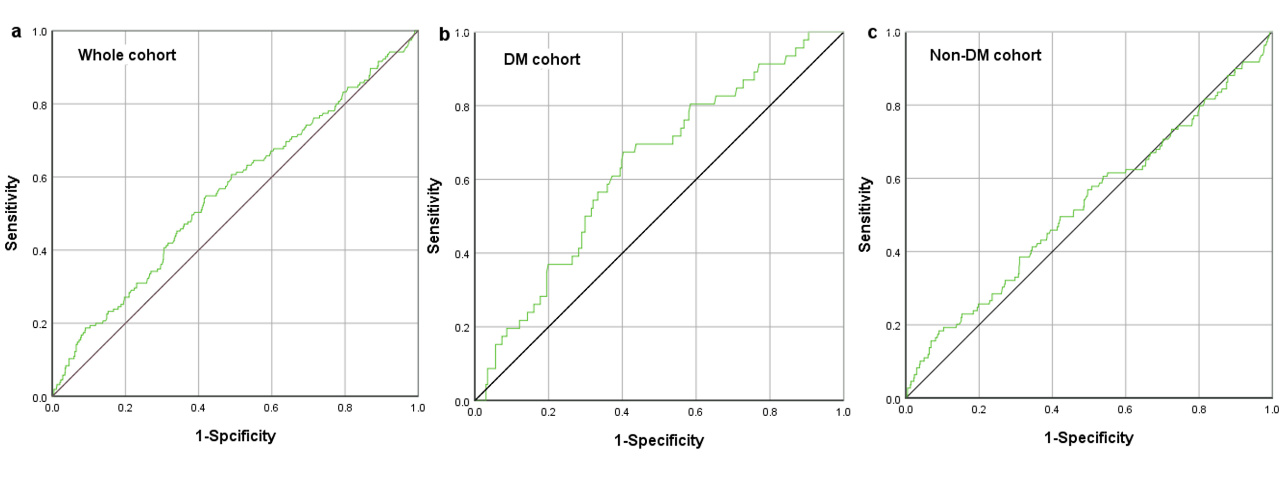
**

**Additional file 2: Fig. S1** Receiver operating characteristic curves of PCSK9 for predicting major adverse cardiovascular events in the whole cohort (**a**), DM patients (**b**) and non-DM patients (**c**). *PCSK9* proprotein convertase subtilisin/kexin type 9, *DM* diabetes mellitus, *Non-DM* non-diabetes mellitus.
